# Mapping Lifestyle Factors in Blood Glucose Variability in Adolescents With Type 1 Diabetes Mellitus: A Pilot Study

**DOI:** 10.1155/pedi/6257886

**Published:** 2026-04-10

**Authors:** Aine Cronin, Therese Dunne, Clodagh O’ Gorman, Alexandra Cremona

**Affiliations:** ^1^ Discipline of Human Nutrition and Dietetics, School of Allied Health, Faculty of Education and Health Sciences, University of Limerick, Limerick, Ireland, ul.ie; ^2^ Department of Paediatrics, University Hospital Limerick, Limerick, Ireland, ul.ie; ^3^ Department of Paediatrics, School of Medicine, University of Limerick, Limerick, Ireland, ul.ie; ^4^ Irish Nutrition and Dietetics Institute, Dublin, Ireland; ^5^ Health Research Institute, University of Limerick, Limerick, Ireland, ul.ie

**Keywords:** adolescents, glycaemic variability, nutrition, physical activity, type 1 diabetes

## Abstract

**Introduction:**

Optimal glycaemic control in adolescents with type 1 diabetes (T1D) is essential to prevent complications but remains challenging due to changing lifestyle behaviours.

**Objective:**

This pilot study aims to assess whether adolescents with T1D in Ireland meet current nutrition and physical activity (PA) guidelines and to explore the impact of nutrition and PA on glycaemic variability (GV).

**Methods:**

Seven adolescents with T1D recorded their PA, diet and blood glucose levels over seven consecutive days. GV was determined using continuous glucose monitoring (CGM) data.

**Results:**

The majority of participants demonstrated low levels of PA, with 72% falling below recommended levels, and 86% consuming excessive amounts of saturated fat. Blood glucose levels were in the very high and high ranges for 23.6% ± 25.2% and 22.6% ± 7.3% of the time, respectively, with only 52.6% ± 21.8% of the time spent within the target range. Although no significant associations were found between PA or nutrition and GV, participants who met or exceeded PA and protein guidelines and consumed less fat exhibited better GV parameters.

**Conclusion:**

Adolescents with T1D in Ireland are not meeting recommended PA and nutrition guidelines and show poor glycaemic control. As a pilot study, the small sample size limited statistical power, but observed trends suggest that adhering to lifestyle recommendations could improve glycaemic control in this population.

## 1. Introduction

Type 1 diabetes (T1D) mellitus is a chronic autoimmune disease leading to destruction of the insulin‐producing pancreatic beta cells and thus to deficiency in insulin secretion [1]. Because of the physiological consequences of high blood glucose levels, regular exogenous insulin delivery becomes necessary to sustain life [2]. Among the various demographic groups affected, the management of adolescents is particularly challenging. In 2021, there were over 1 million children and adolescents aged 0–19 years old with T1D globally, with cases increasing every year [3]. The International Diabetes Federation estimates that there are approximately 3364 Irish children and adolescents aged 0–19 years old living with T1D, with an average incidence rate of 285 new cases diagnosed annually [3]. A recent study found that the incidence rate of T1D in Irish children under the age of 15 is increasing, with 1027 incident cases reported to the Irish Childhood Diabetes National Register in the 3 year period between 2019 and 2021 [4].

Adolescence is a developmental stage characterised by rapid physical, psychological and social changes, resulting in evolving lifestyle behaviours that can significantly influence health outcomes [5]. For adolescents with T1D, this stage adds even more complexity to the management of a chronic condition. The transition from childhood to adolescence involves factors, such as increased independence, heightened peer influence, academic pressures and changing dietary preferences, which often complicate the management of T1D [6, 7]. Compared to other paediatric age groups, adolescents with T1D have poor treatment adherence rates, resulting in suboptimal glycaemic control [8, 9]. This is thought to be associated with the physical and psychological change experienced during this time [10]. Maintaining stable blood glucose levels within the recommended therapeutic target range is essential in preventing acute complications, such as hypoglycaemia or hyperglycaemia, as well as reducing the risk of long‐term complications, including cardiovascular disease, retinopathy, nephropathy and neuropathy [11, 12].

Lifestyle management in the form of exercise and nutrition are important factors in glycaemic management in adolescents with T1D [13]. The International Society for Paediatric and Adolescent Diabetes (ISPAD) states that adolescents with T1D should meet the same activity recommendations as healthy adolescents [14]. Daily moderate physical activity (PA) of at least 60 min or more, with higher intensity exercise and muscle and bone strengthening exercise at least three times weekly is recommended [14]. In addition, from a nutritional perspective, ISPAD recommends the distribution of macronutrients as carbohydrates to be 45%–50% of energy with less than 10% sugar, fat less than 35% of energy with saturated fat less than 10% and protein 15%–20% of energy [15]. However, previous studies have reported that adolescents with T1D are not meeting PA and nutrition guidelines, with many having low levels of activity and high energy intake from fats [16–19].

Despite the recognition of the pivotal role that PA and nutrition play in managing diabetes, there remains a gap in understanding the intricate relationships between these lifestyle factors and blood glucose variability, specifically in adolescents with T1D. Understanding the dynamic nature of these influences is necessary to tailor interventions which optimise blood glucose management, therefore, minimising the risk of complications in this vulnerable population.

The aims of this pilot study are (a) to determine whether adolescents with T1D are meeting the current nutrition and PA guidelines and (b) to determine whether nutrition and PA influence blood glucose variability in adolescents with T1D. This research will analyse the correlations of these factors on glycaemic control, providing a nuanced understanding of how lifestyle choices influence blood glucose fluctuations in this demographic.

## 2. Methods

This was a pilot observational study designed to explore the feasibility of assessing lifestyle behaviours and their association with glycaemic variability (GV) in adolescents with T1D.

### 2.1. Study Design and Recruitment

Ethical approval was granted by the health service (HSE) Mid‐Western Area Research Ethics Committee (REC ref. 035/2023). Adolescents between the ages of 13 and 18 years old with a clinical diagnosis of T1D for 12 months or more were eligible to take part in the study. Participants were required to be using the Dexcom continuous glucose monitoring system (CGM) for continuity of results. Exclusion criteria included having a co‐morbidity affecting cognitive function, and any known co‐existing metabolic disorders.

**Figure 1:**
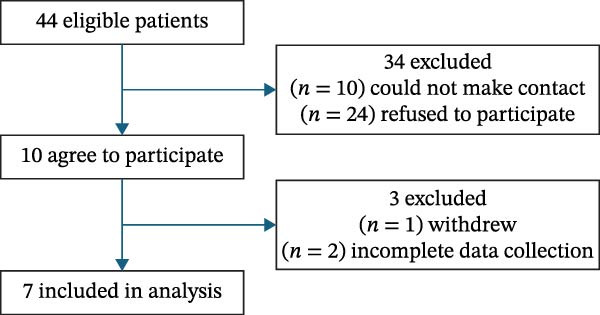
Flowchart of participant recruitment.

Parents/guardians were contacted by the paediatric diabetes team member via email to introduce the study and to provide an information leaflet. The main investigator (ÁC) then met both the parents/guardians and the adolescents at their next clinic visit in University Hospital Limerick to further discuss the study and address any concerns they may have. Participants were informed that participation was entirely voluntary and that their decision to either participate or refuse would not influence their medical care. Sufficient time was given to consider participation. Recruitment was consecutive. In‐person meetings were held with participants and parents/guardians at the University of Hospital Limerick to obtain informed consent and assent and supply the study material and devices. The recruitment process is shown in Figure 1 in the results section.

### 2.2. Data Collection and Analysis

Participants were asked to record their PA, diet (energy intake) and blood glucose levels over seven consecutive days.

Descriptive information for the sample was retrieved from hospital records from the patients most recent hospital appointment. body mass index (BMI) centiles and categories were calculated using the Centers for Disease Control and Prevention (CDC) BMI calculator for children and teens [20].

#### 2.2.1. PA

An accelerometer was provided to participants to measure PA (Fitbit Ace 3). Participants were advised to only remove the Fitbit for sleep (optional) and prior to potential contact with water (such as bathing or swimming). The Fitbit devices provided daily summaries of step count (steps/day) and time spent sedentary, lightly active and moderately–vigorously active (min/day) based on proprietary algorithms. These outputs (min/day) were used to calculate the percentage time spent at each intensity level. Participants were stratified into two groups, based on those who met and those who did not meet the ISPAD recommendation of at least 60 min moderate–vigorous physical activity (MVPA) daily [14], which is the equivalent to 10,000–11,700 steps [21].

#### 2.2.2. Nutrition

Diet was recorded using the diet‐tracking app Libro, developed by the nutrition analysis software Nutritics. Participants self‐recorded their diet on the app for 7 consecutive days, including the type and weight of food consumed and the time of consumption. The software then determines the total daily calorie intake and the breakdown of the three macronutrients. ISPAD guidelines suggest a daily macronutrient distribution of 45%–50% of energy coming from carbohydrates with less than 10% sugar, less than 35% from fat with less than 10% saturated fat and 15%–20% from protein [15].

#### 2.2.3. GV

All participants were using the Dexcom CGM device for the duration of the study. CGM is routinely provided and funded for all children and adolescents with T1D through the national HSE. Using the downloaded CGM data, GV was calculated using the free software EasyGV [22]. Parameters utilised within the analysis were average blood glucose, blood glucose standard deviation (SD), coefficient of variation (CV), mean amplitude of glycaemic excursions (MAGE), mean absolute glucose (MAG), low blood glucose index (LBGI), high blood glucose index (HBGI), mean of daily differences (MODD) and continuous overall net glycaemic action index (CONGA). GV is also shown by percentage of time per day spent with blood sugars in the following ranges: very high, high, in range, low and very low.

#### 2.2.4. Statistical Analysis

Data were analysed using a combination of the software Excel and the Statistical Package for the Social Sciences (IBM SPSS v29.0.2.0). Mean ± SD was used to describe the sample characteristics, and the Shapiro–Wilk test was used to confirm normality. Participants were stratified into different groups to quantify the proportion meeting nutrition and PA recommendations versus those not meeting them. For between‐group comparisons of blood glucose variability, bivariate associations were assessed using the independent samples *t*‐test for normally distributed variables and the Mann–Whitney test for non‐normally distributed variables. To control for multiple comparisons, both Bonferroni and Holm–Bonferroni corrections were applied. For all analyses, *p*  < 0.05 was considered statistically significant.

## 3. Results

### 3.1. Participant Recruitment

Ten adolescents consented to participate in the study (four male/six female). Seven participants were included in the data analysis (two male/five female); one participant withdrew from the study during the data collection period and two participants returned incomplete data that could not be included in the analysis. The recruitment process of participants is shown in Figure 1.

### 3.2. Participant Demographics

The average age of participants was 15 ± 1.2 years (mean ± SD). The average body height was 166 ± 7.8 cm, while weight was 61.4 ± 4.5 kg. The average BMI and BMI centiles were 22.3 ± 2.2 and 69.1 ± 20.8, respectively. Five participants were classed as healthy based on their BMI centile, while two were classed as overweight. All participants were receiving insulin therapy during the study with one participant using pens and six using insulin pumps. Participant characteristics are shown in Table 1.

**Table 1:** Participant characteristics (mean ± SD).

Gender, *N* (%)	
Male	2 (28)
Female	5 (72)
Age (years)	15 ± 1.2
Body height (cm)	167 ± 7.9
Body weight (kg)	61.8 ± 4.6
BMI (kg/m^2^)	22.2 ± 2.3
BMI centile	68.3 ± 22.3
BMI category, *N* (%)	
Healthy (5^th^–85^th^ centile)	5 (72)
Overweight (85^th^–95^th^ centile)	2 (28)
Insulin, *N* (%)	
Pens	1 (14)
Pump	6 (86)

**Figure 2:**
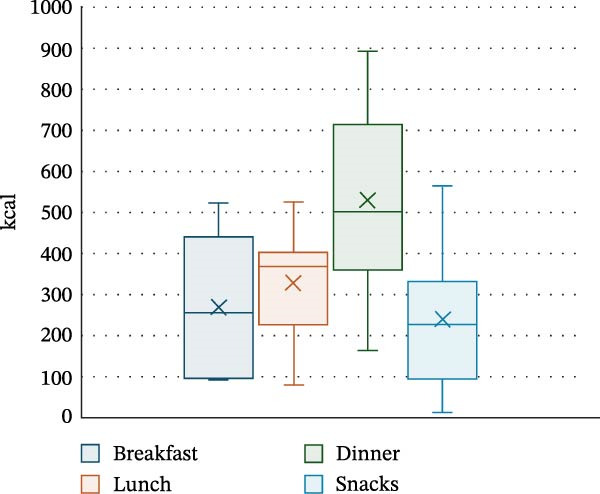
Energy distribution (kcal) throughout the day based on meals.

**Figure 3:**
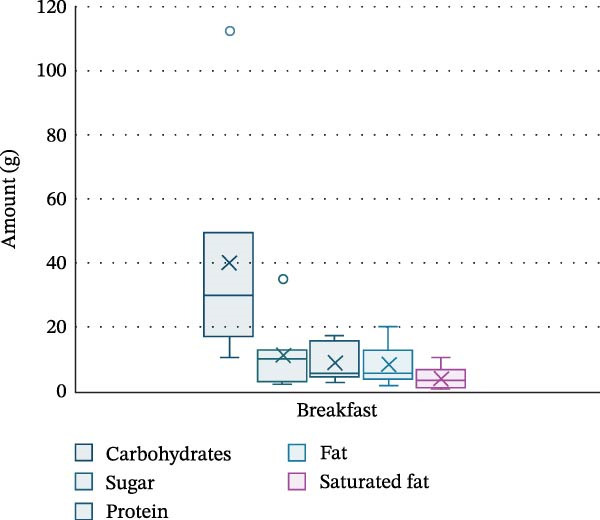
Macronutrient distribution for breakfast.

**Figure 4:**
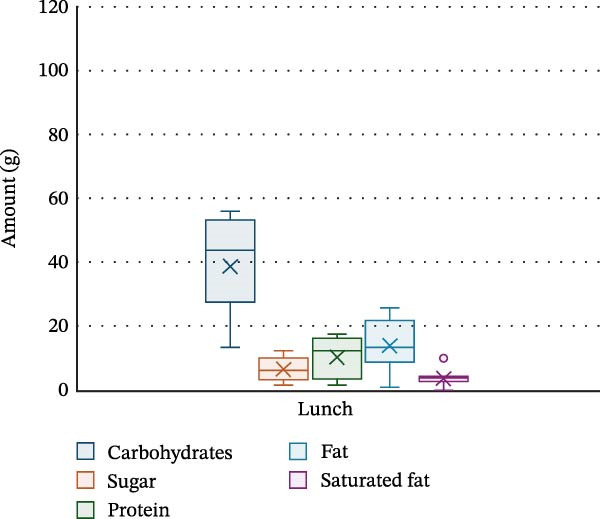
Macronutrient distribution for lunch.

**Figure 5:**
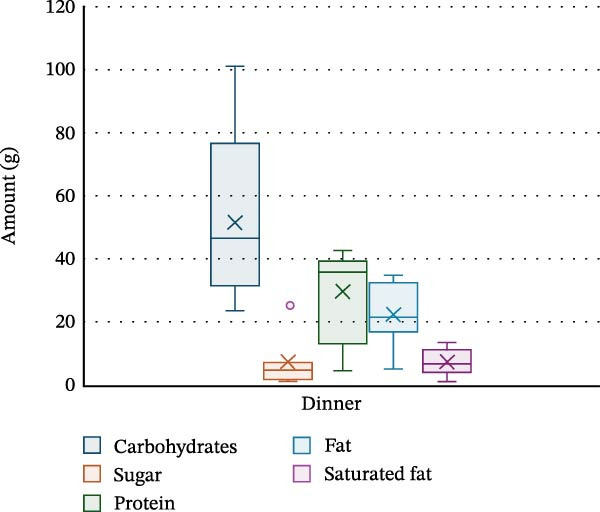
Macronutrient distribution for dinner.

**Figure 6:**
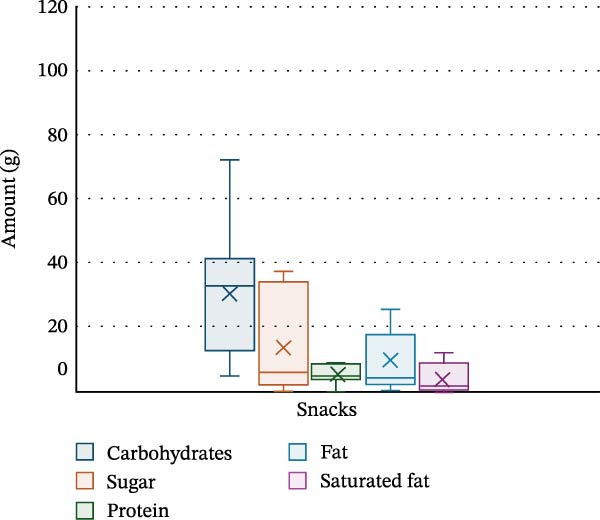
Macronutrient distribution for snacks.

PA and nutrition parameters are shown in Table 2. The mean number of steps taken by participants per day was 8830 ± 4263, with 81.4% ± 5.1% of time per day spent sedentary and only 2.6% ± 2.1% spent moderately–vigorously active. The mean energy intake of participants was 1368.7 ± 521.1 kcal per day, with carbohydrates contributing 47.8% ± 7.2% of energy intake, fats 36.1% ± 4.5% and saturated fat 13.2% ± 3% (20.1 ± 9.2 g). Dinner was the meal with the highest calorie intake (530.5 ± 241.8 kcal), with carbohydrate, protein, fat and saturated fat intake being the highest at this meal (52 ± 27.6 g, 30 ± 14.7 g, 22.4 ± 10.5 g and 7.3 ± 4.3 g, respectively). Snacks were the main source for sugar in this cohort (14.3 ± 15.5 g). Energy and macronutrient distribution across meals is shown in Figures 2–6.

**Table 2:** Physical activity and nutrition parameters (*n* = 7).

Physical activity	Average number of steps (steps/day)		8830 ± 4263
	Sedentary	(min/day)	1171 ± 73.9
		%	81.4 ± 5.1
	Lightly active	(min/day)	231.3 ± 47.9
		%	16 ± 3.3
	Moderately–vigorously active	(min/day)	37.4 ± 30.3
		%	2.6 ± 2.1

Nutrition	Energy intake	(kcal/day)	1368.7 ± 521.1
		(kJ/day)	5747.4 ± 2190.3
	Carbohydrate intake	(g/day)	162.3 ± 72.7
		%	47.8 ± 7.2
	Of which sugars	(g/day)	40.2 ± 23.2
		%	11.8 ± 4.3
	Fat intake	(g/day)	55.3 ± 21.5
		%	36.1 ± 4.5
	Of which saturated fat	(g/day)	20.1 ± 9.2
		%	13.2 ± 3
	Protein intake	(g/day)	55.2 ± 20.6
		%	16 ± 4.4

Table 3 shows the breakdown of participants who were below or met/exceeded the recommendations for the PA and nutrition parameters. Only two (28%) out of the seven participants met or exceeded the PA recommendation of 60 min MVPA per day. Four (57%) participants met or exceeded the carbohydrate recommendation of 45%–50%, while four (57%) participants also met or exceeded the sugar recommendation of ≤10%. Three (43%) participants met or exceeded the fat recommendation of ≤35%, while six (86%) exceeded the saturated fat recommendation of ≤10%. Five (72%) participants met or exceeded the protein recommendation of 15%–20%.

**Table 3:** Physical activity and nutrition target levels (*n* = 7).

Category	Median (IQR)	Min–max	Recommended	*N* (%) below recommendation	*N* (%) that met/exceeded recommendation
Physical activity	32 (46)	9–94	60	5 (72)	2 (28)
Carbohydrates	48.2 (13.4)	39.6–58.6	45–50	3 (43)	4 (57)
Sugar	14.0 (7.0)	6.1–17.4	≤10	3 (43)	4 (57)
Fat	33.2 (6.4)	32.2–44.1	≤35	4 (57)	3 (43)
Saturated fat	13.7 (2.4)	7.4–17.2	≤10	1 (14)	6 (86)
Protein	16.3 (6.7)	9.2–22.1	15–20	2 (28)	5 (72)

*Note:* carbohydrates (%/day); fat (%/day); physical activity (min/day); protein (%/day); saturated fat (%/day); sugar (%/day).

**Figure 7:**
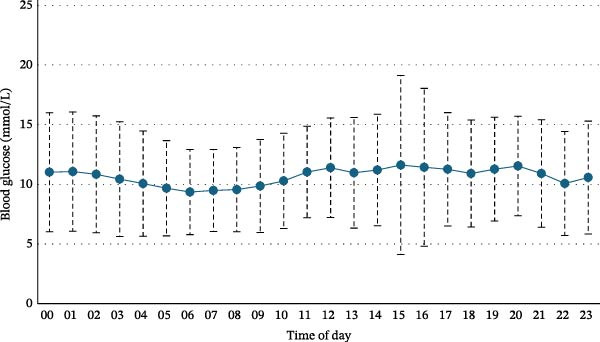
Mean 24‐h blood glucose levels for the cohort (*n* = 7).

Figure 7 shows the mean 24‐h blood glucose fluctuations for the cohort, while Table 4 shows the GV parameters for the whole group. Participants spent a large portion of the day above the very high and high threshold for blood glucose levels, 23.6% ± 25.2% and 22.6% ± 7.3%, respectively. On average, only 52.6% ± 21.8% of time was spent within range of the target blood glucose levels. Time spent in the low and very low ranges was minimal (<1% each). The average blood glucose level for the sample was 10.5 ± 2.4 mmol/L, with substantial variability (blood glucose SD 3.7 ± 0.9 mmol/L; CV 36.4 ± 7.7%). Advanced GV metrics (MAGE, CONGA, LBGI, HBGI, MODD and MAD) are reported in Table 4.

**Table 4:** Glycaemic variability for whole sample (*n* = 7).

Parameter	Quantity
Time per day spent in very high (>13.9 mmol/L) (%)	23.6 ± 25.2
Time per day spent in high (>10.0 mmol/L) (%)	22.6 ± 7.3
Time per day spent in range (3.9–10.0 mmol/L) (%)	52.6 ± 21.8
Time per day spent in low (<3.9 mmol/L) (%)	0.7 ± 0.8
Time per day spent in very low (<3.0 mmol/L) (%)	0.6 ± 0.5
Average blood glucose	10.5 ± 2.4
SD	3.7 ± 0.9
CV	36.4 ± 7.7
MAGE	9.7 ± 3.8
CONGA	9.1 ± 2.3
LBGI	2.5 ± 1.4
HBGI	13.9 ± 7.7
MODD	3.6 ± 0.6
MAG	4.2 ± 1.1

*Note:* Average blood glucose (mmol/L). CV, blood glucose coefficient of variation (%); SD, blood glucose standard deviation (mmol/L).

Abbreviations: CONGA, continuous overall net glycaemic action (mmol/L); HBGI, high blood glucose index; LBGI, low blood glucose index; MAG, mean absolute glucose (mmol/L) (mean ± SD); MAGE, mean amplitude of glycaemic excursions (mmol/L); MODD, mean of daily differences (mmol/L).

### 3.3. Effect of PA and Nutrition on GV

Table 5 show the differences in GV between those who met or exceeded the PA and nutrition recommendations and those who did not. Although there were no significant associations calculated, there were some interesting differences between the groups.

**Table 5:** Differences in glycaemic variability between those who met and exceeded physical activity and nutrition recommendations and those who did not.

Measure of glyaemic variability	Physical activity (recommended 60 min MVPA daily)			Carbohydrates (recommended 45%–50% of daily calorie intake)			Sugar (recommended ≤10% of daily calorie intake)		
	Met/exceeded recommendation (*n* = 2)	Below recommendation (*n* = 5)	*p*‐Value	Met/exceeded recommendation (*n* = 4)	Below recommendation (*n* = 3)	*p*‐Value	Met/exceeded recommendation (*n* = 4)	Below recommendation (*n* = 3)	*p*‐Value
Avg. blood glucose	8.7 ± 1.0	11.2 ± 2.5	0.121	9.7 ± 1.5	11.6 ± 3.3	0.162	9.7 ± 1.5	11.6 ± 3.3	0.170
SD	3.3 ± 0.3	3.9 ± 1.0	0.226	3.9 ± 1.2	3.6 ± 0.1	1.0	3.8 ± 1.2	3.7 ± 0.2	0.432
CV	38.0 ± 1.1	35.7 ± 9.3	0.379	39.6 ± 6.9	32.1 ± 7.6	0.115	38.7 ± 7.2	33.2 ± 8.6	0.197
MAGE	8.4 ± 1.4	10.3 ± 4.5	0.571	10.7 ± 5.0	8.4 ± 1.0	0.234	10.1 ± 5.3	9.2 ± 0.8	0.400
CONGA	7.4 ± 1.0	9.8 ± 2.4	0.122	8.4 ± 1.6	10.1 ± 3.1	0.199	8.3 ± 1.6	10.2 ± 3.0	0.163
LBGI	3.0 ± 1.5	2.3 ± 1.4	0.279	2.1 ± 1.0	3.1 ± 1.8	0.400	2.3 ± 1.4	2.7 ± 1.6	0.380
HBGI	8.8 ± 2.4	15.9 ± 8.4	0.157	11.3 ± 4.5	17.3 ± 10.7	0.173	10.8 ± 4.3	17.9 ± 10.3	0.132
MODD	3.1 ± 0.2	3.9 ± 0.6	0.082	3.7 ± 0.8	3.6 ± 0.4	0.392	3.4 ± 0.6	4.0 ± 0.6	0.141
MAG	3.4 ± 0.1	4.5 ± 1.1	0.119	3.8 ± 0.9	4.7 ± 1.3	0.164	3.9 ± 0.9	4.7 ± 1.3	0.193
Time/day in very high	9.5 ± 3.5	29.2 ± 28.4	0.199	15.3 ± 10.6	34.7 ± 37.5	0.234	13.8 ± 10.1	36.7 ± 36.0	0.193
Time/day in high	18.5 ± 7.8	24.2 ± 7.3	0.200	22.8 ± 6.7	22.3 ± 9.6	0.474	24.0 ± 7.9	20.7 ± 7.6	0.299
Time/day in range	70.5 ± 9.2	45.4 ± 21.6	0.095	60.3 ± 15.0	42.3 ± 28.5	0.162	60.5 ± 14.8	42.0 ± 28.4	0.153
Time/day in low	1.0 ± 1.4	0.6 ± 0.5	1.000	1.0 ± 0.8	0.3 ± 0.6	0.400	1.0 ± 0.8	0.3 ± 0.6	0.400
Time/day in very low	0.5 ± 0.7	0.6 ± 0.5	0.857	0.8 ± 0.5	0.3 ± 0.6	0.400	0.8 ± 0.5	0.3 ± 0.6	0.400

Measure of glyaemic variability	Fat (recommended ≤35% of daily calorie intake)			Saturated fat (recommended ≤10% of daily calorie intake)			Protein (recommended 15%–20% of daily calorie intake)		
	Met/exceeded recommendation (*n* = 3)	Below recommendation (*n* = 4)	*p*‐Value	Met/exceeded recommendation (*n* = 6)	Below recommendation (*n* = 1)	*p*‐Value	Met/exceeded recommendation (*n* = 5)	Below recommendation (*n* = 2)	*p*‐Value
Avg. blood glucose	11.6 ± 3.3	9.7 ± 1.5	0.162	10.6 ± 2.6	10.0	0.423	10.6 ± 2.8	10.3 ± 1.7	0.452
SD	3.6 ± 0.1	3.9 ± 1.2	1.000	3.7 ± 1.0	3.9	0.571	3.5 ± 0.3	4.3 ± 1.9	0.346
CV	32.1 ± 7.6	39.6 ± 6.9	0.115	35.9 ± 8.3	39.0	0.373	34.8 ± 6.5	40.3 ± 11.9	0.857
MAGE	8.4 ± 1.0	10.7 ± 5.0	0.234	9.7 ± 4.2	9.9	0.571	8.5 ± 1.1	12.9 ± 7.4	0.278
CONGA	10.1 ± 3.1	8.4 ± 1.6	0.199	9.2 ± 2.5	8.8	0.448	9.1 ± 2.6	9.1 ± 1.9	0.496
LBGI	3.1 ± 1.8	2.1 ± 1.0	0.400	2.4 ± 1.5	3.1	0.341	2.8 ± 1.4	1.6 ± 1.1	0.156
HBGI	17.3 ± 10.7	11.3 ± 4.5	0.173	13.9 ± 8.4	13.4	0.479	14.5 ± 8.8	12.2 ± 6.2	0.379
MODD	3.6 ± 0.4	3.7 ± 0.8	0.392	3.5 ± 0.5	4.5	0.063	3.6 ± 0.7	3.7 ± 0.8	0.484
MAG	4.7 ± 1.3	3.8 ± 0.9	0.164	4.2 ± 1.2	4.4	0.424	4.4 ± 1.1	3.8 ± 1.3	0.289
Time/day in very high	34.7 ± 37.5	15.3 ± 10.6	0.234	24.2 ± 27.5	20.0	1.000	26.2 ± 29.3	17.0 ± 15.6	1.000
Time/day in high	22.3 ± 9.6	22.8 ± 6.7	0.474	22.0 ± 7.8	26.0	0.328	21.2 ± 8.3	26.0 ± 2.8	0.241
Time/day in range	42.3 ± 28.5	60.3 ± 15.0	0.162	52.7 ± 23.9	52.0	0.490	51.2 ± 25.2	56.0 ± 17.0	0.409
Time/day in low	0.3 ± 0.6	1.0 ± 0.8	0.400	0.7 ± 0.8	1.0	0.360	0.8 ± 0.8	0.5 ± 0.7	0.339
Time/day in very low	0.3 ± 0.6	0.8 ± 0.5	0.400	0.5 ± 0.5	1.0	0.571	0.6 ± 0.5	0.5 ± 0.7	1.000

*Note:* Data expressed as mean ± SD. Average blood glucose (mmol/L); CV, blood glucose coefficient of variation (%); SD, blood glucose standard deviation (mmol/L). As only 1 participant met the recommendation for saturated fat, all glycaemic variability parameters are constant when the saturated fat recommendation is met. Participants who exceeded the fat recommendation were the same individuals who were below the carbohydrate recommendation.

Abbreviations: CONGA, continuous overall net glycaemic action (mmol/L); HBGI, high blood glucose index; LBGI, low blood glucose index; MAG, mean absolute glucose (mmol/L); MAGE, mean amplitude of glycaemic excursions (mmol/L); MODD, mean of daily differences (mmol/L).

Those who met or exceeded the PA recommendation had noticeably lower measurements for average blood glucose (8.7 ± 1.0 mmol/L vs. 11.2 ± 2.5 mmol/L, *p* = 0.121), spent more time per day within range of the blood glucose target levels (70.5% ± 9.2% vs. 45.4% ± 21.6%, *p* = 0.095) and had lower MODD, MAG and time spent above the very high threshold for blood glucose levels (Table 5).

For nutrition, those who met or exceeded the recommendation for carbohydrates had lower average blood glucose levels (9.7 ± 1.5 mmol/L vs. 11.6 ± 3.3 mmol/L, *p* = 0.162) and time above the very high threshold (15.3% ± 10.6% vs. 34.7% ± 37.5 %, *p* = 0.234), respectively). Additionally, time per day spent within range of the blood glucose target levels were noticeably higher in these individuals (60.3 ± 15.0 vs. 42.3 ± 28.5, *p* = 0.162).

Of note, those who were below the recommendation for sugar consumption had higher average blood glucose levels (11.6 ± 3.3 mmol/L vs. 9.7 ± 1.5 mmol/L, *p* = 0.170) and spent more time per day above the very high threshold for blood glucose levels (36.7% ± 36.0% vs. 13.8% ± 10.1%, *p* = 0.193). However, they spent less time per day within range of the blood glucose target levels (42.0 ± 28.4 vs. 60.5 ± 14.8, *p* = 0.153).

Those who were below the fat consumption recommendation had lower average blood glucose levels (9.7 ± 1.5 mmol/L vs. 11.6 ± 3.3 mmol/L, *p* = 0.162) and spent more time within range of the blood glucose target levels (60.3% ± 15.0% vs. 42.3% ± 28.5%, *p* = 0.162).

One participant consumed within the saturated fat recommendation. This individual did not statistically different between measurements of those who met or exceeded the recommendation.

For protein consumption, those who met or exceeded the recommendation spent less time per day above the high recommendation for blood glucose levels (21.2% ± 8.3% vs. 26.0% ± 2.8%, *p* = 0.241). However, they also had higher measurements for LBGI (2.8 ± 1.4 vs. 1.6 ± 1.1, *p* = 0.156).

Both the Bonferroni and Holm–Bonferroni corrections were applied to account for multiple comparisons. These corrections produced no significant results, indicating that the observed differences did not reach statistical significance when controlling for multiple comparisons.

## 4. Discussion

This pilot study aimed to explore the correlation between lifestyle factors, specifically PA and nutrition and GV in adolescents with T1D. The study found that a significant portion of participants did not meet recommended PA levels or dietary guidelines, and this non‐compliance was associated with poorer glycaemic control.

### 4.1. Adherence to PA and Nutrition Recommendations

Analyses revealed that most participants did not meet the recommended guidelines for either PA or dietary intake. These findings are congruent with previous research indicating that adolescents with T1D are not meeting the daily PA and dietary intake recommendations to achieve optimal health [16, 23]. Only two participants exceeded the recommended 60‐min MVPA per day. Other studies in Ireland have previously highlighted the poor adherence to PA in Irish adolescents with T1D [16, 24], despite being a key element for improving glycaemic control and quality of life [25]. Adherence to nutritional recommendations in terms of macronutrient distribution was also suboptimal among the participants. While 43% were below the carbohydrate threshold, 43% exceeded the fat intake threshold and 57% exceeded the sugar threshold. The high consumption of sugar through snacks shows a need for healthier alternatives. As reported in other international studies [23, 26, 27], we observed an overly high consumption of saturated fat, with six of the seven participants exceeding the recommended daily intake. This is of particular concern due to the resulting poor glucose control and the increased risk of cardiovascular disease [28]. Data analysed from the ‘Growing Up in Ireland’ longitudinal study found that Irish children living with chronic illnesses are more likely to have lower levels of PA and poorer diets in comparison to their peers with no chronic illnesses [29]. Although data analysed in the study were collected between 2007 and 2008, the results are alarmingly similar, suggesting that there may not have been significant improvements in the last 16 years.

### 4.2. GV

GV refers to the fluctuations in blood glucose levels over a given period, and it is a critical parameter in managing T1D. High GV is associated with both short‐term risks, such as hypoglycaemia and hyperglycaemia, and long‐term complications, including cardiovascular disease, retinopathy, nephropathy and neuropathy [30]. Therefore, understanding the factors that influence GV is vital for improving the management of T1D in adolescents. It has been widely reported that poor glycaemic control is an issue in adolescents with T1D [31–33]. The findings in this study are reflective of this. In this pilot study, several measures of GV were used, including average blood glucose, MAGE, CONGA, LBGI, HBGI, MODD and MAG, with all parameters showing considerable deviations from the target ranges. When assessing the short‐term within‐day GV, MAGE is considered as the ‘gold standard’ [34], while MODD is generally accepted as a standard indicator for assessing the between‐days GV [35]. This cohorts’ measurements for both MAGE (9.7 ± 3.8 mmol/L) and MODD (3.6 ± 0.6 mmol/L) indicates high GV with high day‐to‐day variability and frequent fluctuations. This is concerning as long‐term blood sugar fluctuations can result in major health complications that harm vital organs such as the heart, kidneys and eyes [30]. Although time in range is not technically an index of GV, it provides valuable information about the quality of glycaemic control [36], with a higher time in range is associated with better GV [36]. Participants spent 23.6% and 22.6% of time in the very high and high blood glucose ranges, respectively, with only 52.6% of time spent within the target range, again suggesting poor glycaemic control, and indicating that hyperglycaemia is a common issue. Hyperglycaemia is known to increase during adolescence and is commonly attributed to poor communication and changes experienced during the transition from paediatric to adult care [37]. This variability is concerning as it increases the risk of both acute and long‐term complications in T1D [12].

### 4.3. Effect of PA and Nutrition on GV

A recent systematic review reported that low levels of PA and extensive sedentary behaviour is a contributing factor to poor glycaemic control in adolescents with T1D [38]. Unfortunately, this study did not reveal any statistically significant associations between meeting or not meeting PA guidelines and GV. Despite this, there were notable trends suggesting that better PA levels may be linked to improved glycaemic control. For example, participants who met or exceeded the PA recommendation tended to exhibit lower average blood glucose levels and spent more time within the target range for blood glucose levels. This, along with lower measurements for HBGI, MAG and CONGA, suggests more stable and consistent glucose levels and a lower risk of hyperglycaemia in those who met or exceeded the PA recommendations. Previous studies have reported that MVPA is associated with better glycaemic control resulting in fewer extreme fluctuations, and so, fewer hypo‐ and hyperglycaemic episodes [39–42].

There is a paucity of research investigating the relationship between dietary intake and glycaemic outcomes. Although there were no statistically significant associations observed between dietary intake and GV, there were intriguing differences in glycaemic outcomes between those who met or exceeded the recommendations for certain macronutrients and those who did not. For instance, participants who met or exceeded the 45%–50% carbohydrate intake recommendation tended to have lower average blood glucose levels and spent more time within target range for blood glucose levels, suggesting better glycaemic control. They also exhibited lower measurements for HBGI, and MAG, indicating that those who met or exceeded the carbohydrate intake recommendation have less GV, with lower exposure to high blood glucose levels. In contrast to this, Cherubini et al. [43] found that when adolescents with T1D consumed 40%–44% of carbohydrates instead of the recommended 45%–50%, the probability of spending more than 70% of time within target range of blood glucose levels was significantly higher. Additionally, Monzon et al. [44] found that adolescents with high carbohydrate intake experienced greater postprandial GV. Low carbohydrate diets are a common approach in T1D management as they have been associated with greater glycaemic control [45]. However, this approach is not recommended for children and adolescents with T1D as it does not ensure the sufficient daily caloric intake, increasing the risk of stunted development [46].

For protein, those who met or exceeded the 15%–20% intake recommendation spent less time above the high threshold for blood glucose levels, however, spent more time below the low threshold. They also had higher measurements for LBGI and MAG, which suggests that those who met or exceeded the protein intake recommendation exhibit greater GV and have a greater risk of hypoglycaemia. Similarly, previous studies have found that high protein intake in children and adolescents with T1D is associated with higher postprandial glycaemic excursions [47–50]. However, in contrast, Monzon et al. [44] found that adolescents with high protein intake experienced lower postprandial GV. Furthermore, Cherubini et al. [43] reported that participants who spent more than 70% of time within range of blood glucose levels consumed a higher percentage of protein, while participants with a protein consumption of less than 15% experienced less than 70% of time in range. Those who were below the ≤10% sugar intake recommendation exhibited higher average blood glucose levels and spent less time within the target range for blood glucose levels and more time above the very high threshold, suggesting that those who consume below the sugar intake recommendation present with greater short‐term GV and a higher risk of hyperglycaemia.

High fat intake has been linked to increased postprandial glycaemic excursions and hyperglycaemia [47, 48, 50], with high saturated fat intake leading to poorer glycaemic control and increased risk of cardiovascular disease [28, 51]. Results from this study are congruent with these findings. Those who were below the ≤35% fat intake recommendation presented with lower average blood glucose levels and spent more time within target range for blood glucose levels. They also had lower measurements for CONGA, HBGI and MAG, suggesting that those who consumed below the fat recommendation had less short‐term GV with a lower risk of hyperglycaemia. One participant consumed below the ≤10% saturated fat intake recommendation. This individual presented as very similar to those who consumed above the recommendation. The only notable difference was that they had a higher measurement for MODD, suggesting greater day‐to‐day variability and less stable glucose control in comparison to those who consumed above the saturated fat recommendation. Although this assumption is contradictory to previous studies exploring the effect of saturated fat on blood glucose control [28, 51, 52], the unbalanced stratification along with the overall small sample size could have affected the analysis and reduced the possibility of significant associations.

### 4.4. Future Research

The results of this pilot study suggest several implications for clinical practice and future research. The results underscore the importance of comprehensive lifestyle management in adolescents with T1D. Despite the lack of significant associations, the trends observed in the data warrant further research with a larger cohort to examine the relationship between lifestyle factors and GV more comprehensively. A larger cohort would allow for stratification into more nuanced groups based on adherence to lifestyle guidelines, for example, those who met the recommendation, those who were below the recommendation, and those who exceeded the recommendation. The suboptimal adherence to the PA and nutrition recommendations is another important avenue to explore. Further research is warranted to examine the facilitators and barriers adolescents experience in relation to exercising and healthy eating. Investigating factors, such as personal, social and environmental influences could provide valuable insights. Previous studies have identified parental and peer support, self‐motivation, competing leisure activities, self‐management and knowledge as factors that influence adolescents’ motivation for PA and healthy eating [53–56]. However, there is a lack of research into this in an Irish setting. Understanding these reasons can inform the development of targeted interventions to improve adherence to PA and nutritional guidelines and, consequently, glycaemic control in this population. Healthcare providers should focus on personalised strategies that consider the unique developmental and psychological challenges faced by adolescents [10].

### 4.5. Strengths and Limitations

Several limitations must be acknowledged when interpreting the findings of this study. The small sample size (*n* = 7) and short duration of the study (7 days) limited the statistical power of the analysis and may have limited the ability to detect significant associations. With an already small potential pool for recruitment, further difficulty was experienced with many potential recruits reporting the anticipated time commitment as a major barrier to participation. Additionally, human error, particularly in self‐recording dietary intake, and device error, such as issues with the Fitbit and Dexcom system, may have introduced inaccuracies into the data. Recruitment bias may also have affected this study. Research has shown that certain personality traits influence an individual’s decision to volunteer or not [57]. Consequently, adolescents who consented to participate in the study in comparison to those who declined might be better educated about T1D and more determined to impact greater change. Future research should involve larger cohorts and longer monitoring periods to validate these preliminary findings. Moreover, exploring psychosocial factors and incorporating behavioural interventions could provide deeper insights into improving adherence to lifestyle recommendations in this population.

## 5. Conclusion

This pilot study has highlighted that adolescents with T1D in Ireland are not meeting the recommended PA and dietary intake guidelines as set forth by the ISPAD [14, 15], and also exhibit poor glycaemic control. While not statistically significant, it also provides valuable insights into the relationship between the lifestyle factors and GV in Irish adolescents with T1D, warranting further research to confirm and expand on these findings. By elucidating the relationships between PA, dietary habits and glycaemic control, future studies can inform the development of targeted interventions to optimise health outcomes in this vulnerable population.

**Nomenclature**
BMI: Body mass index
CGM: Continuous glucose monitoring
CONGA: Continuous overall net glycaemic action index
CV: Coefficient of variation
GV: Glycaemic variability
HBGI: High blood glucose index
ISPAD: The International Society for Paediatric and Adolescent Diabetes
LBGI: Low blood glucose index
MAG: Mean absolute glucose
MAGE: Mean amplitude of glycaemic excursions
MODD: Mean of daily differences
MVPA: Moderate–vigorous physical activity
PA: Physical activity
SD: Standard deviation
T1D: Type 1 diabetes.

## Author Contributions

**Aine Cronin:** recruitment, data collection, formal analysis, investigations, writing – original draft, visualisation. **Therese Dunne:** recruitment, manuscript review. **Clodagh O’ Gorman:** manuscript review. **Alexandra Cremona:** conceptualisation, resources, writing – review and editing, supervision, project administration.

## Funding

This work was funded by a seed funding grant from the Health Research Institute, Faculty of Education and Health Science, Univeristy of Limerick, Ireland. Awarded to the principle investigator Alexandra Cremona.

## Ethics Statement

Ethical approval was granted by the HSE Mid‐Western Area Research Ethics Committee (REC Ref. 035/2023).

## Conflicts of Interest

The authors declare no conflicts of interest.

## Data Availability

The data that support the findings of this study are openly available in Zenodo at https://doi.org/10.5281/zenodo.13749141, Reference Number 10.5281/zenodo.13749140.
